# Supplementary figures and images for: Evidence-based recommendations on storing and handling specimens for analyses of insect microbiota
Source: PeerJ. 2015 Aug 18;3:e1190. doi: 10.7717/peerj.1190 (PMC4548535; doi:10.7717/peerj.1190)

### Butterfly

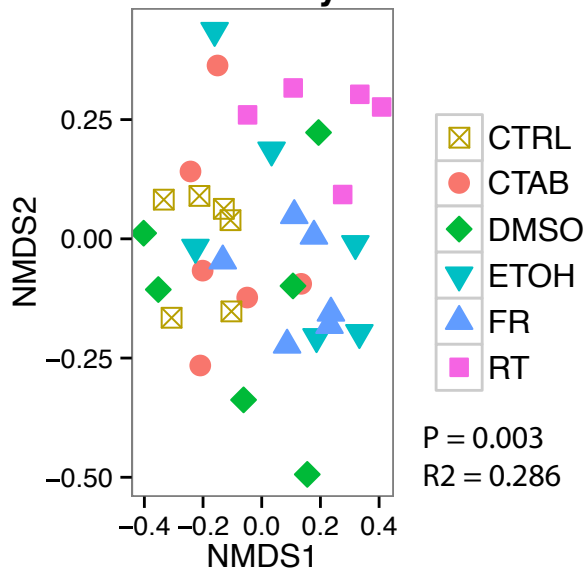

### Grasshopper

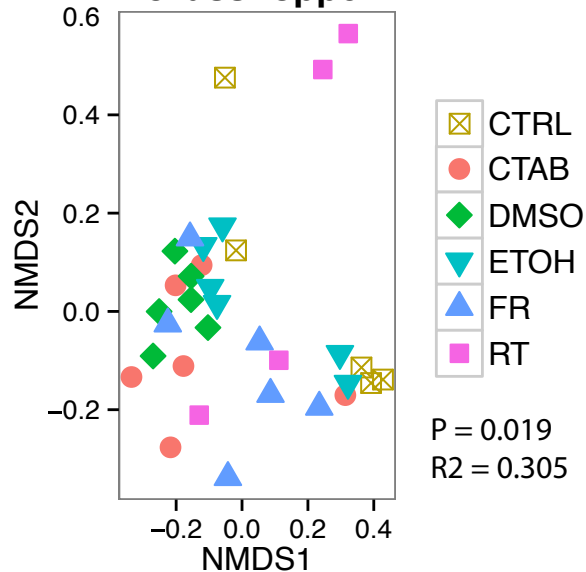

### Bee

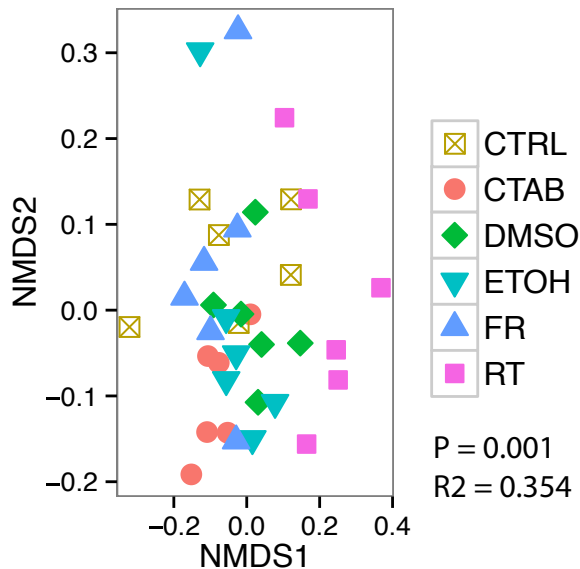

### Beetle

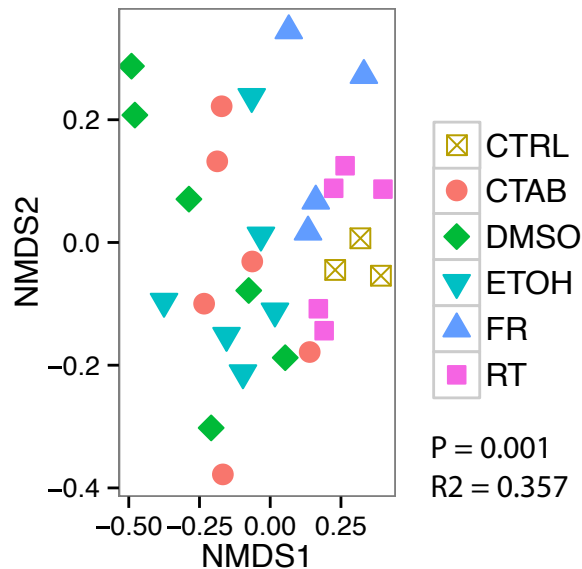

Supplement: Figure S1 — Ordinations of samples showing differences in bacterial community structure for each insect species across the storage methods. Results from PERMANOVA tests are given below the legends. FR, freezer; RT, room temperature; ETOH, ethanol; CTRL, controls. [file peerj-03-1190-s001.pdf]

FR vs CTRL

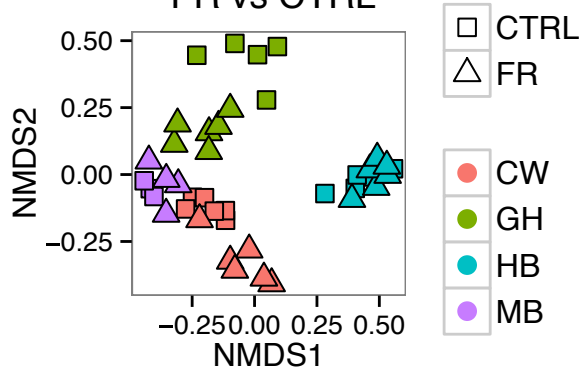

DMSO vs CTRL

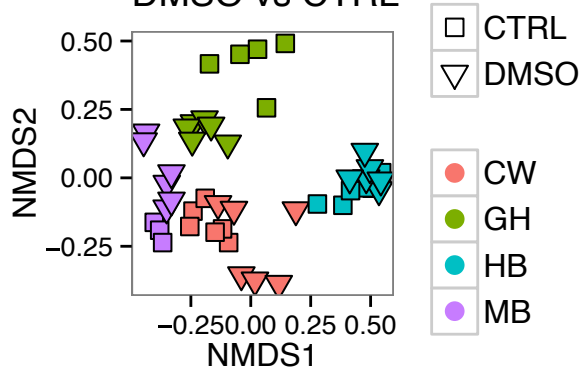

RT vs CTRL

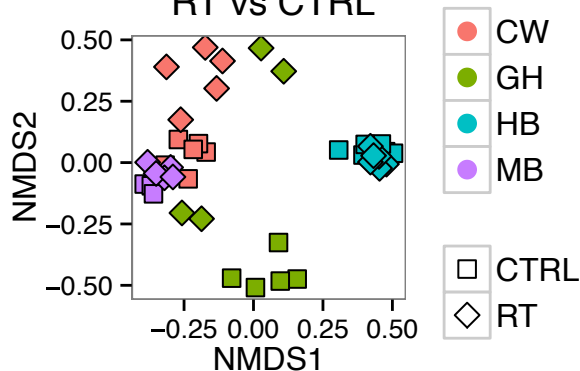

CTAB vs CTRL

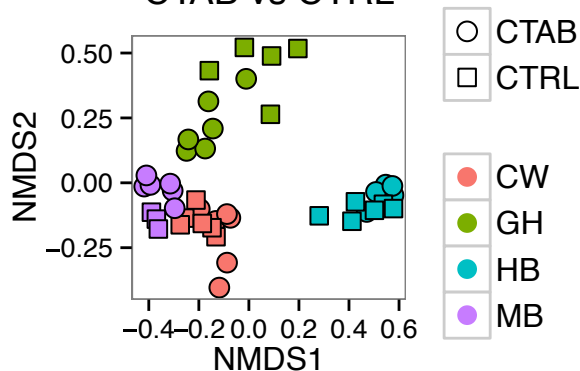

ETOH vs CTRL

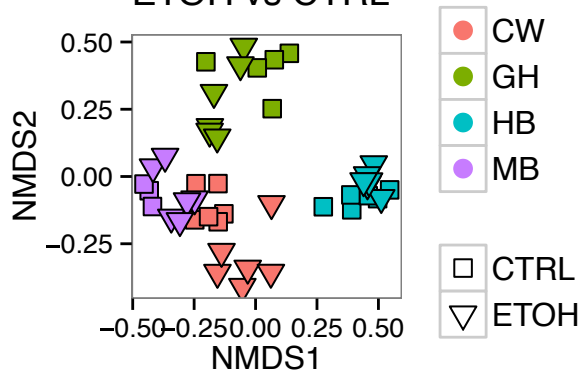

Supplement: Figure S2 — Ordinations comparing each storage method and the controls for all four insect species, highlighting that differences in bacterial community structure between the insect species exceed any potential storage effects in all cases. Statistical results are given in Table 1. CW, cabbage white butterfly; HB, honey bee; MB, Mexican bean beetle; GH, grasshopper; FR, freezer; RT, room temperature; ETOH, ethanol; CTRL, controls. [file peerj-03-1190-s002.pdf]

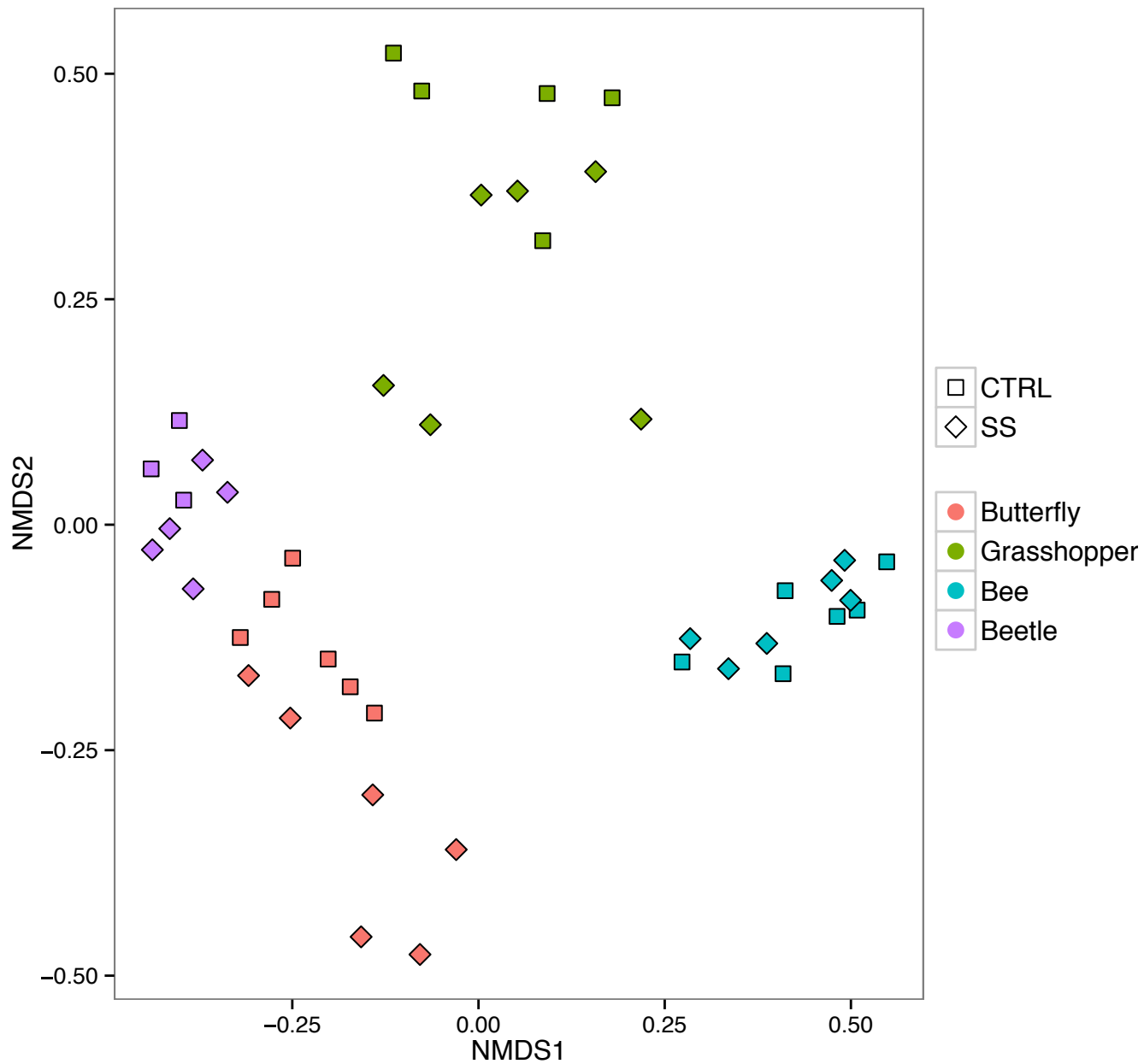

Supplement: Figure S3 — Ordination of control versus surface-sterilized insects, colored by insect species. SS, surface sterilized; CTRL, controls. [file peerj-03-1190-s003.pdf]
