# Supplementary material for: Evidence-based recommendations on storing and handling specimens for analyses of insect microbiota
Source: PeerJ. 2015 Aug 18;3:e1190. doi: 10.7717/peerj.1190 (PMC4548535; doi:10.7717/peerj.1190)
Supplement: Table S2 — Dominant bacterial genera whose relative abundances differed significantly between a storage method and the control (P < 0.05 after FDR correction). Dashes indicate that no genera from that species/storage combination differed. Where bacteria could not be classified to genus, the family classification is shown. CW, cabbage white butterfly; HB, honey bee; MB, Mexican bean beetle; GH, grasshopper. [file peerj-03-1190-s005.docx]

| **Species** | **-20°C** | **ETOH** | **CTAB** | **DMSO** | **Room Temperature** |
| --- | --- | --- | --- | --- | --- |
| CW | - | - | - | - | < *Acinetobacter*  < *Pseudomonas*  *<* Pseudomonadaceae  < *Alicyclobacillus* |
| HB | - | - | > *Bifidobacterium*  > *Lactobacillus* | - | < *Lactobacillus*  < Acetobacteraceae |
| MB | - | - | - | - | - |
| GH | - | - | - | > *Sphingomonas*  > *Neisseria* | > *Enterococcus* |
